# A framework for conducting economic evaluations alongside natural experiments

**DOI:** 10.1016/j.socscimed.2018.11.032

**Published:** 2019-01

**Authors:** Manuela Deidda, Claudia Geue, Noemi Kreif, Ruth Dundas, Emma McIntosh

**Affiliations:** a Health Economics & Health Technology Assessment, Institute of Health & Wellbeing, University of Glasgow, 1 Lilybank Gardens, Glasgow, G12 8RZ, United Kingdom; b Centre for Health Economics, University of York, Heslington, York, YO10 5DD, United Kingdom; c MRC/CSO Social and Public Health Sciences Unit, University of Glasgow, 200 Renfield Street, Glasgow, G2 3QB, United Kingdom

**Keywords:** Natural experiments, Guidance, Economic evaluation, Checklist, Population health interventions

## Abstract

Internationally, policy makers are increasingly focussed on reducing the detrimental consequences and rising costs associated with unhealthy diets, inactivity, smoking, alcohol and other risk factors on the health of their populations. This has led to an increase in the demand for evidence-based, cost-effective Population Health Interventions (PHIs) to reverse this trend. Given that research designs such as randomised controlled trials (RCTs) are often not suited to the evaluation of PHIs, Natural Experiments (NEs) are now frequently being used as a design to evaluate such complex, preventive PHIs. However, current guidance for economic evaluation focusses on RCT designs and therefore does not address the specific challenges of NE designs. Using such guidance can lead to sub-optimal design, data collection and analysis for NEs, leading to bias in the estimated effectiveness and cost-effectiveness of the PHI. As a consequence, there is a growing recognition of the need to identify a robust methodological framework for the design and conducting of economic evaluations alongside such NEs. This paper outlines the challenges inherent to the design and conduct of economic evaluations of PHIs alongside NEs, providing a comprehensive framework and outlining a research agenda in this area.

## Highlights

• NEs differ from RCTs, requiring tailored guidance on conducting economic evaluation.
• Ignoring NEs' specific traits in PHI economic evaluation may lead to sub-optimal design and bias.
• First paper to provide guidance for conducting economic evaluation alongside NEs.

## 1 Introduction

Evaluating the effectiveness and cost-effectiveness of Population Health Interventions (PHIs) has become an area of increasing interest for researchers and decision makers. The UK's National Institute for Health and Care Excellence (NICE) recently updated their methods guidance to account for the specific requirements of PHIs economic evaluation (e.g. broader cost-benefit framework)(NICE, 2012, 2014). In line with this, and very much in tune with the increased attention being paid to the broader topic of ‘The Economics of Prevention’ (Merkur et al., 2013) there has been an increase in both applied and methodological research on the economic evaluation of PHIs (Carter et al., 2009; Edwards et al., 2013; Greco et al., 2016; McDaid et al., 2015; Tudor Edwards and McIntosh, 2018; Weatherly et al., 2009).

Given the complex nature of many PHIs (Byford and Sefton, 2003; Smith and Petticrew, 2010) in addition to the complexity of systems within which they are delivered (Shiell et al., 2008), the identification of a credible causal effect is a key methodological issue. Randomised controlled trials (RCTs) have traditionally been regarded as the ‘gold standard’ methodology for estimating the causal effects of PHIs (Bonell et al., 2011). The attractiveness of RCTs stems mainly from randomisation, resulting in a ‘closed’ system, where researchers can control exposure of participants to the intervention or to the control group, eliminating or controlling those factors which have been retained to be potential confounders (McDonnell et al., 2009). This results in the most important advantage of RCTs, namely protection against selection bias due to observed and unobserved differences between treatment groups.

The researcher's control within an RCT design also extends to data collection, which can be tailored to directly measure outcomes and costs deemed to be relevant for the economic evaluation, as well as identify potential confounders, that may need to be adjusted for, to increase the precision of estimates.

However, randomisation might be neither practical, nor ethical for PHIs, which are often not amenable to standard evaluation methodology (Craig et al., 2008; Manca and Austin, 2008). Furthermore, randomisation *per se* does not guarantee unbiased estimates of average treatment effects (ATEs) in every setting, (for example because of practical failures to balance treatment and control groups or non-adherence to treatment), thus ruling out the superiority of RCT with respect to other methodologies to estimate the causal effect (Deaton and Cartwright, 2018). In such situations, natural experiments (NEs) can provide a viable alternative ‘vehicle’ for evaluation and economic evaluation.

NEs can be defined as “naturally occurring circumstances in which subsets of a population have different level of exposure to a supposed causal factor, in a situation resembling an actual experiment where human subjects would be randomly allocated to groups”(Last et al., 2001). Unlike RCTs, NEs have a non-randomised design, where assignment to intervention cannot be controlled by the researcher, instead the ‘intervention’ and ‘control’ groups are distinguished with respect to observable and unobservable factors that may be related to the outcome of interest (Deeks et al., 2003). Non-randomisation represents a threat to internal validity, and a credible source of exogenous variation (e.g. random geographical and temporal variations in the availability of the intervention) is required to be able to identify the true causal effect (Meyer, 1995). In NEs, since the researcher cannot control the source of randomness, the use of statistical designs and methodologies to deal with any resulting selection bias is advocated.

Despite arguably lower internal validity however NEs have the potential for a higher external validity and higher “real world” relevance than RCTs (Baltussen et al., 1999). For these reasons, this methodology is increasingly being adopted for the evaluation of PHIs (Craig et al., 2017). Existing guidance for designing, conducting and reporting economic evaluations alongside RCTs (Drummond et al., 2015; Glick et al., 2007; Husereau et al., 2013) do not address the specific challenges of economic evaluation alongside NEs. In addition, existing literature on conducting evaluations of NEs is scant and focuses on effectiveness only (e.g. Craig et al. (2017)). A related literature explores specific statistical and econometric issues inherent to non-randomised studies, for economic evaluations (Kreif et al., 2013a; Rovithis, 2013), but does not explore the specific issues related to PHIs.

Following general RCT guidelines does not account for the specific challenges of NEs in the design and conduct of economic evaluation of PHIs such as identifying appropriate sources of linked data from the early stage of design, encompassing methodologies to reduce selection bias into a cost-effectiveness framework and incorporating externalities and spatial spillovers using observational data sources. This could lead to a biased estimation of the causal effect of the intervention thus lowering the quality of evidence on effectiveness and cost-effectiveness.

Drawing on equivalent standards available for reporting and presenting the results of economic evaluation alongside RCTs (Husereau et al., 2013), this paper formally contrasts established methods and guidance for conducting cost-effectiveness analysis (CEA) alongside RCTs in a bid to emphasise key differences with NEs. In doing so, we review existing literature, identify gaps in existing methodological research and provide a framework to guide the researcher from the early design phase through to conducting the economic evaluation, including a set of recommended best practices: the selection of multiple comparisons groups; identification of the most appropriate sources of data to conduct the economic evaluation; appropriate sensitivity analysis using different comparison groups/different methodologies; use of decision modelling; inclusion of an economic logic model. From this exercise we develop a critical appraisal checklist with the aim to outline the specific requirements for designing and conducting economic evaluations alongside NEs. This checklist can also be used as a practical tool for improving the quality and consistency of economic evaluations alongside NEs.

## 2 Methods

A targeted scoping review of existing literature and reporting guidelines was carried out to identify the key methodological issues inherent to the economic evaluation of PHIs alongside NEs (details are provided in the online appendix, A2).

This literature review is accompanied by a critical review of the most common reporting guidelines: CHEERS (Husereau et al., 2013), STROBE (Von Elm et al., 2014) and TREND (Des Jarlais et al., 2004). CHEERS summarizes a comprehensive set of well-established best practices covering economic evaluations alongside RCTs. STROBE and TREND, while not considering economic evaluation, cover non-randomised PHIs and observational studies framework, respectively.

All three guidelines were critically reviewed in terms of their applicability and relevance for economic evaluations in NEs and synthesised to identify best practices for reporting economic evaluations in NEs. Every item listed in the three guidelines has been critically assessed in relation to existing literature on NEs and PHIs e.g. (Craig et al., 2017; Drummond et al., 2007; Lorgelly et al., 2010; Petticrew et al., 2005; Weatherly et al., 2009) and good design, conduct and reporting standards for RCTs (Drummond et al., 2015; Glick et al., 2007; Petrou and Gray, 2011). Only the items which were identified to be relevant to evaluate PHIs alongside NEs were retained, while items specific to RCTs or to other frameworks (e.g. observational studies exploring the association between a risk factor and a health outcome) were discarded. An iterative approach was used to adapt and refine existing reporting standards to our specific NE focus.

The output of the scoping review was a critical appraisal checklist of good practice for designing and conducting economic evaluations alongside NEs. The checklist comprises a set of recommendations, corresponding to ten broad items, which have been described in relation to the challenges for the economic evaluation alongside NEs with the view of emphasising key differences with respect to RCTs. Concrete examples of economic evaluations alongside NEs from the literature, when available, have been used throughout the paper to exemplify adherence to this suggested set of best practices. The checklist has been critically reflected upon by applying it to the economic evaluation of a case study, the Healthy Start Voucher (HSV) scheme (Dundas et al., 2014b), whose evaluation and economic evaluation has been funded by the NIHR Public Health Research. A description of the HSV case study and a practical application of the checklist to this specific case study have been provided in the supplementary appendix A1.

## 3 Results

The scoping review resulted in the identification of four broad sets of literature: i)research considering specific challenges in the economic evaluation of PHIs (e.g. outcome measurement; intersectoral costs and consequences; equity), but not specific to NEs, e.g. (Drummond et al., 2007; Lorgelly et al., 2010; Smith and Petticrew, 2010; Weatherly et al., 2009) ii) methodological research describing specific issues related to the reduction of selection bias inherent to non-randomised studies in a cost-effectiveness framework, (e.g Kreif et al. (2013a); Manca and Austin (2008)); iii)research describing the benefits and challenges of using NEs in PHIs, but not specifically referring to economic evaluation (e.g. Craig et al. (2017); Petticrew et al. (2005)); iv)case studies of economic evaluation of PHIs in a non-randomised context, (e.g.(Alfonso et al. (2015); Leyland et al. (2017)). The analysis of reporting guidelines, complemented with a focussed revision of the literature, has revealed a complete lack of comprehensive guidance on how to design economic evaluations of PHIs alongside NEs. Table 1 lists items identified from this review.

**Table 1:** Reporting guidelines.

	Item differs between RCTs and NEs	Item is reported in the guideline		
		**CHEERS**	**TREND**	**STROBE**
*Background/objectives*	Yes	Yes	Yes	Yes
*Target population*	Yes	Yes	Yes	Yes
*Sample size*	Yes	No	Yes	Yes
*Subgroup definition and analysis*	Yes	Yes	Yes	Yes
*Setting and location*	Yes	Yes	Yes	Yes
*Study perspective*	Yes	Yes	No	No
*Comparators*	Yes	Yes	Yes	No
*Time horizon/length of follow-up*	Yes	Yes	Yes	Yes
*Data sources/measurement*	Yes	No	No	Yes
*Choice of health outcomes*	Yes	Yes	Yes	Yes
*Measurement and valuation of preference based outcomes*	Yes	Yes	No	No
*Estimating resources and costs*	Yes	Yes	No	No
*Currency, price, date and conversion*	No	Yes	No	No
*Analytical methods*	Yes	Yes	Yes	Yes
*Methods to address confounding*	Yes	No	Yes	Yes
*Variables (outcomes, exposure, predictors, potential confounders, effect modifiers)*	Yes	No	No	Yes
*Bias and methodology to correct bias*	Yes	No	Yes	Yes
*Missing data imputation methods*	No	Yes	Yes	Yes
*Study parameters*	No	Yes	No	No
*Incremental costs and outcomes*	No	Yes	No	No
*Characterising uncertainty*	No	Yes	No	Yes
*Characterising heterogeneity*	No	Yes	Yes	Yes
*Discount rate*	No	Yes	No	No

As shown in Table 1, RCTs and NEs differ for most items, however for items which do not differ, we refer readers to existing CHEERS guidelines (Husereau et al., 2013). With many of the items listed inTable 1 being correlated (e.g. the choice of an appropriate time horizon relates to data availability, outcomes and costs) these were grouped into ten categories pertinent to the design and conduct of economic evaluations alongside NEs. Building on these ten items, a critical appraisal checklist was developed addressing specific requirements for designing and conducting economic evaluations alongside NEs (Table 2). The following sections describe the items presented in the checklist, highlighting challenges, item-specific differences with the RCT framework and providing practical examples.

**Table 2:** Checklist for the economic evaluation of PHIs alongside NEs.

Item description	Has the study complied with the item?		
	YES	No	NA
**1. Data sources and measurement**			
**1.1 The data used and the reason(s) why it has been chosen has been identified, stated and described in relation to:**			
1.1.1 all relevant intersectoral outcomes and costs being captured			
1.1.2 implementation of the chosen statistical design			
**1.2 The application to routinely collected administrative data has been done on time to avoid delays in conducting economic evaluations (e.g. due to bureaucratic procedures, anonymization, privacy and confidentiality requirements).**			
**1.3 The study recognize and address attrition and missing data and its consequences for the health economics analysis (bias)**			
**1.4 The study recognize and address measurement errors (e.g. due to discrepancies between the timing of the intervention and period of data availability) and its consequences for the health economics analysis (bias)**			
**2. Setting and location**			
**2.1 Setting and location are stated and explained in relation to social and political priorities**			
**2.2 The source of secondary data that best meets the economic evaluation needs in terms of setting and location has been stated**			
**2.3 Concurrent interventions have been:**			
2.3.1 Identified			
2.3.2 Tackled with appropriate statistical analysis (e.g. robustness checks; subsample analysis)			
**2.4 Potential spillovers/externalities effects have been:**			
2.4.1 Identified through the usage of an economic evaluation logic model			
2.4.2 Addressed through appropriate sensitivity analysis			
**3. Choice of comparators**			
**3.1 The choice of comparators is justified in relation to reduction of selection bias due to non-randomisation, the unit of assignment (individual or aggregate) and data availability**			
**3.2 The existence of potential spillovers/crossovers has been considered in the choice of comparators**			
**3.3 Multiple intervention/control groups have been used to examine sensitivity of the economic evaluation to multiple sources of bias**			
**4. Subgroups**			
**4.1 If equity concerns are included in the economic evaluation, subgroups are defined in relation to distributional concerns**			
**4.2 Potential behavioural responses (e.g. ‘nudge effects’), have been identified and measured**			
**5. Outcome**			
**5.1 An economic evaluation model mapping routinely collected intermediate outcomes to QALYs has been developed, using additional evidence from systematic reviews to identify utility values.**			
**5.2 An economic evaluation framework such as CCA, CBA or MCDA has been chosen and justified**			
**6. Costs**			
**6.1 Costing has been done considering a societal perspective**			
**6.2 When unit cost data associated to a specific resource use are not available, a decision rule (e.g. usage of the average unit cost of the most frequently used service) is explained and justified.**			
**6.3 When specific categories of resource use are not publicly available a decision rule is explained and justified.**			
**6.4 The opportunity cost of transfer payments (i.e. transfer of resources from the government to beneficiaries, with a null net impact on society) has been identified and measured**			
**7. Time horizon**			
**7.1 Linked data are adequate to capture the presence of long term effects**			
**7.2 Appropriate discount rates, in line with the most up to date guidance have been applied**			
**8. Inclusion of a logic model**			
**8.1. A logic model has been developed, and it addresses:**			
8.1.1 Time horizon(e.g. effects that would 'carry over' after the intervention ended)			
8.1.2 possible subgroups effect			
8.1.3 externalities and spillovers			
**9. Analytical methods**			
**9.1 The researchers have justified the source of variation in the exposure to the intervention, choosing a design and a statistical approach which is appropriate in relation to that source of variation.**			
9.1.1 If the study is a before after design frequent measurements of data on long pre-treatment time periods have been collected			
**9.2 Multiple statistical designs have been employed to examine the sensitivity of economic evaluation to multiple sources of bias**			
**9.3 The list of potential confounders has been presented**			
**9.4 Causal effects have been interpreted considering potential contaminating policies**			
**9.5 The interpretation of the estimated cost-effectiveness is in line with the estimated parameter**			
**9.6 The methodologies to reduce selection bias have been incorporated into an economic evaluation framework, considering health economics-specific challenges (i.e. skewed outcome and cost data, correlated outcome and cost data).**			
**10. Uncertainty and sensitivity analysis**			
**10.1 All sources of uncertainty have been identified using appropriate methods (e.g. probabilistic sensitivity analysis; tornado diagrams)**			
**10.2 Cost-effectiveness results according to the different analytical choices have been reported**			
**10.3 Sensitivity analysis has been done in relation to:**			
10.3.1 assumptions made in relation to unit cost			
10.3.2 potential spillovers			
10.3.3 comparators			
10.3.4 different designs			
10.3.5 econometric methodology chosen			
10.3.6 unobserved confounding			
10.3.7 transfer payments and administrative costs			

### 3.1 Category 1: data sources and measurement

It is usually unfeasible or unpractical in a NE setting to conduct individual patient-level data collection tailored to the specific requirements for a health economic evaluation. Hence, using multiple, sometimes linked, observational data sources (e.g. surveys, registries, administrative records or census data) will likely represent standard practice. The choice of observational data sources should be justified in relation to their capability to capture the broad spectrum of intersectoral cost and consequence impacts often associated with PHIs (item 1.1.1). Furthermore, suitability of data sources in relation to the chosen statistical approaches to reduce selection bias (e.g. longitudinal study for a before/after approach; adequacy of sample size; availability of suitable instruments for Instrumental Variable (IV) approach) should be explicitly justified (item 1.1.2).

Leyland et al. (2017) evaluated the Health in Pregnancy (HiP) grant, a universal conditional cash transfer, introduced for women reaching the 25th week of pregnancy, with the aim of improving birth weight and other birth outcomes. They used several data sources including a maternity and neonatal database, morbidity records, mother's obstetric records to capture relevant outcomes (e.g. birthweight; gestation at booking, booking before 25 weeks) and costs (e.g. hospitalizations and delivery costs) for all registered birthsacross the pre-intervention, intervention and post-intervention period. The period covered by the linked data was sufficiently long to exploit temporal variation, and compare outcomes in periods immediately after the introduction of the HiP grant with those periods before its introduction and after its withdrawal.

The use of administrative data can often represent an advantage over primary data collection, by overcoming issues of loss to follow-up and low response rates, which can represent considerable challenges when evaluating PHIs targeted towards disadvantaged populations (Petticrew et al., 2005). Using routinely collected administrative data is also likely to reduce measurement error and mitigate challenges of recall bias inherent in survey data. Administrative data arguably provides fairly precise and objective estimates of healthcare usage and costs incurred by the NHS, the healthcare provider, society and the individual (Husain et al., 2012). Furthermore, observational data may be available for a longer time span than data collected alongside short follow up RCTs. This allows the researcher to track the identified target population both prospectively and retrospectively (Husain et al., 2012). Despite these advantages, there are challenges such as bureaucratic procedures, anonymization, privacy and confidentiality requirements which may cause delays in data availability (item 1.2).

In addition to handling attrition and missing values (item 1.3), the researcher has to address issues which are more specific to observational data such as measurement errors in confounding variables, due to discrepancies between the timing of the intervention and period of data availability (item 1.4). Whilst the methodology employed to handle missing data (e.g. multiple imputation) is often reported in economic evaluations of PHIs alongside NEs (e.g. (Dundas et al., 2014a; Leyland et al., 2017), only few examples address the possible bias arising from specific issues related to observational data. For example, Alfonso et al. (2015) address the potential bias stemming from different data sources (household survey and health facility register) used to capture the main outcome pre and post intervention.

### 3.2 Category 2: setting and location

Unlike RCTs, where the researcher decides the target population and location, in a NE framework, setting and location are fixed and determined by the intervention or policy being evaluated. The target population may be defined by a policy-maker (who sets eligibility criteria of the programme), potentially influenced by social or political priorities. For example, a high maternal mortality rate motivated the intervention evaluated by Alfonso et al. (2015), while concerns related to the health of mothers of low socioeconomic background lead to the design of the HiP evaluated by (Leyland et al., 2017). The researcher needs to state these social and political priorities (item 2.1).

The use of secondary data sources typically allows the assessment of effectiveness and cost effectiveness of the intervention over larger sample size (often the entire population) than would be available in an RCT, but these sources might also restrict the choice of target population. For example, the choice of Scottish mothers even if the policy has been implemented throughout the UK as target population for the economic evaluation of HiP (Leyland et al., 2017) was driven not only by the availability of high quality routine data, but also by the specific characteristics of Scotland in terms of concentration of deprivation (Leyland et al., 2017). Hence, the choice of a target population that best meets the evaluation needs is important (item 2.2).

Identifying who is affected by the PHI in a NE framework is often not straightforward, especially in relation to the complexity of PHIs, which usually involve several interacting components (Craig et al., 2008). Moreover in a real-world setting, several concurrent, interacting policy interventions may be in place at the same time making it challenging to separately identify the effects of the different policies. Such concurrent interventions should be identified (item 2.3.1), and measured by employing appropriate statistical analyses (e.g. robustness checks; subsample analysis) (item 2.3.2). In the HiP study, the introduction of the ban on smoking in enclosed public spaces in Scotland was considered as a potentially contaminating policy and an analysis that restricted pre-treatment periods to after the introduction of the smoking ban was carried out. (Leyland et al., 2017).

The possibility of externalities (when producing or consuming a good/intervention causes a positive or negative impact on third parties), spillovers (wider health benefits), and ‘cross-over’ effects or ‘contamination’ (individuals from the control group migrating towards the intervention group) while also possible in RCT settings, poses an increased challenge for NEs (Petticrew et al., 2005). First, any treatment ‘contamination’ can lead to misclassification of individuals into intervention and control groups, Second, even if the intervention and controls groups are well-defined, the effect a PHI may affect several groups, or extend to individuals or areas, which are beyond the scope of the intervention. While general frameworks to incorporate spill-over effects within economic evaluations have been developed (e.g. Al-Janabi et al. (2011)), these might require additional data collection outwit the NEs framework which typically makes use of existing observational data sources.

The existence of cost and consequence spill-overs and externalities could be identified using an economic evaluation logic model, (described in Section 3.8) (item 2.4.1), and addressed through a sensitivity analysis that would include a broad set of multi-sectoral costs and outcomes into the analysis (item 2.4.2).

### 3.3 Category 3: comparators

The choice of intervention and control group is a major challenge in non-randomised studies (Petticrew et al., 2005). In an evaluation of NEs, this needs to be aligned with the choice of methodology to reduce selection bias due to non-randomisation, the unit of assignment (individual or aggregate) and data availability (item 3.1).

It is important to choose the control group which maximises internal validity, increasing a researcher's ability to attribute differences in outcomes and costs to the intervention, and not to other confounding factors. In this regards, the existence of spill-overs and contamination, should be identified in the early stages of study design, in order to identify the most appropriate sources of secondary data (item 3.2). Hence, the use of multiple intervention/control groups is recommended to examine the sensitivity of the economic evaluation to multiple sources of bias (Craig et al., 2012; Meyer, 1995) (item 3.3).

### 3.4 Category 4: subgroups

Since many PHIs are often directed towards the reduction of health inequalities (Cookson, 2016) identification of subgroups within an economic evaluation of PHIs alongside NEs could be informed by considerations of equity, in addition to efficiency considerations (item 4.1). In the case of HiP, given the potential of such an intervention to have a greater effect in deprived subgroups, a subgroup analysis by level of deprivation was a key component of the evaluation (Leyland et al., 2017).

Considerations of equity in economic evaluations can be achieved by identifying appropriate subgroups, incorporating value judgments or employing methods that explicitly incorporate equity in the decision making process such as Distributional Cost-Effectiveness analysis (DCEA) (Asaria et al., 2016) or extended cost-effectiveness analysis (Verguet et al., 2015).

Methods which specifically address equity concerns, such as DCEA, are preferred to subgroup analysis if there is potential that the intervention generates an unfair health distribution. However, these methods might be more burdensome in terms of data and computational requirement and may not be feasible when using observational data.

Identification of subgroups would also allow researchers to disentangle potential behavioural responses, such as ‘nudge effects’ that may arise alongside the intervention. While such nudge effects are rarely investigated alongside economic evaluations, they could be used for developing more effective PHIs. It is recommended that researchers complement the traditional economic evaluation methods with the additional of behavioural economic insights by identifying and measuring the existence of potential nudge effects within the PHI (item 4.2).

### 3.5 Category 5: outcomes

Within prospectively designed economic evaluations alongside RCTs researchers typically include a preference based measure of outcome such as the EuroQol EQ-5D (EuroQol Group, 2015) to facilitate calculation of Quality Adjusted Life Years (QALYs). Furthermore, measures that capture spillover QOL effects (e.g. to family members, carers etc.) such as the Carer Experience Scale (CES) (Al-Janabi et al., 2011) can be included. However, without researcher input in the design of outcomes, preference-based outcomes may not be routinely collected, relying on intermediate outcomes. As such, researchers typically focus on the causal effect of the program on intermediate outcomes (e.g. birth weight in the HiP (Leyland et al., 2017). These could subsequently be mapped to generic health measures such as QALYs or Disability Adjusted Life Years (DALYs) using a decision analytical model (Briggs et al., 2006)(item 5.1).

A focus on unidimensional outcomes may be too narrow in PHIs where a battery of multi-sector outcomes may be relevant for inclusion, using a broader societal perspective including benefits to patients, carers and the whole society. For example, PHIs aimed at improving infant mental health result in long term improvements in infant health, educational attainment and employment prospects (Deidda et al., 2018)). This justifies collecting a wide range of outcomes, and the use of broader evaluation frameworks such as cost-benefit analysis (CBA) (Tudor Edwards and McIntosh, 2018), cost-consequence analysis (CCA) (as recommended by the NICE public health economic evaluation guidance) or multi-criteria decision analysis (Marsh et al., 2016) (MCDA). Specifically, MCDA would facilitate the identification and measurement of a plethora of outcomes which are relevant to decision makers, weighting and valuing each of them with methods such as discrete choice experiments (DCEs).

While these methods can be also used in a RCT framework to complement the primary cost-utility analysis-, an increased use of available secondary data in NE's is likely to justify further use of CBA, CCA and MCDA (item 5.2).

### 3.6 Category 6: estimating resources and costs

A societal perspective is recommended best practice for economic evaluations of PHIs (NICE, 2012, 2014), given the inter-sectoral costs often associated with PHIs (item 6.1).

Routinely collected data may not allow for the identification of specific resource use data; e.g. individuals may be asked about hospital length of stay, without any detail on specialty or Health Related Group (HRG). Conditional on the available information from several sources (administrative data, published reports and literature) we recommend that the researcher employs a decision rule for such proxy valuation such as using the average unit cost of the most frequently used service or choosing the maximum/minimum among a set of available unit costs (items 6.2 and 6.3). The choice should reflect the compilation of a standard ‘average’ unit cost.

Transfer payments (transfer of resources from the government to the beneficiaries with null net impact on society) will not be included, even when a broad societal perspective is considered, and the impact of the intervention on how resources are distributed is not taken into account (Byford et al., 2003). However, it may still be informative to identify and measure the opportunity cost or ‘benefits forgone’ of these payments in view of the value attached by the society to the redistribution of wealth in sensitivity analyses (item 6.4).

### 3.7 Category 7: time horizon

The choice of a time horizon ought to account for the presence of long term effects that may ‘carry over’ after an intervention ended. This is particularly relevant for PHIs, where the outcomes of such ‘preventive’ interventions may arise in the future. Unlike RCTs, where the length of time horizon is often constrained (Manca and Austin, 2008), and long term effects can only be identified using extrapolation through decision modelling, NE designs may facilitate a longer time horizon for data collection. This would allow data to capture the, often elusive, long term impacts of PHIs which may be incorporated into a long term decision analytical model, developed following best practices for such complex PHIs (Squires et al., 2016).

To this end, while effects carrying over after the intervention end are not specific to NEs, stating suitability of linked data to capture these effects within a NE framework is strongly recommended in order to adequately take these effects into account.

If it is likely that an intervention has an effect that would ‘carry over’ after an intervention ends, the time horizon should be adjusted to take these effects into account, and should be reflected in the choice of adequate data. If the available data do not allow to follow-up for a time span that would capture such effects, or if the information about long term outcomes of the intervention is not present, the use of external sources is recommended (item 7.1).

In the event of longer term outcomes, discount rates in line with most recent guideline (NICE, 2014) should be applied to costs and benefits (item 7.2).

### 3.8 Logic models and economic evaluations

Within the complex framework of PHIs and NEs, an economic evaluation logic model (NICE, 2014) represents a useful tool to describe anticipated causal pathways and inter-relationships of resource use and outcomes, providing guidance the choice of data collection identifying the behaviour change induced by the intervention, the factors that exert influence on program effectiveness and cost-effectiveness at different levels (individual, social, group level)(e.g. (Deidda et al., 2018).

**Fig. 1:**
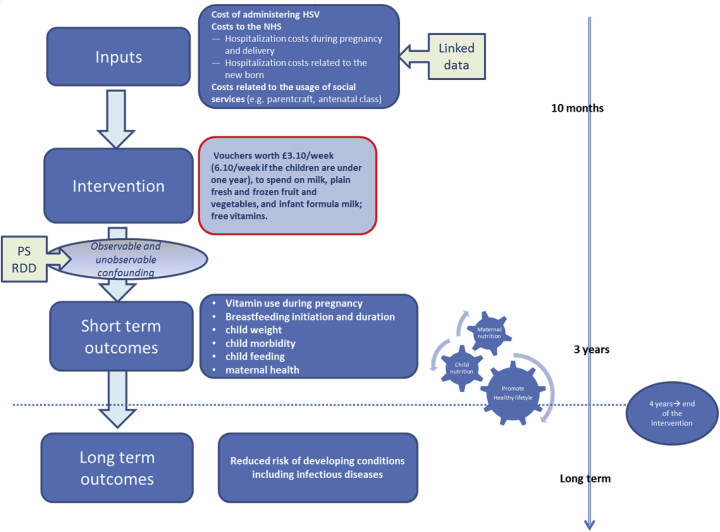
Health Economics logic models.

Despite its role in depicting complexity of PHIs evaluated alongside NEs, thus potentially guiding the researcher from the early stages of design, the economic evaluation logic model does not currently represent standard practice. Fig. 1 shows an illustrative example of the logic model for the HSV project.

### 3.9 Category 8: analytical methods

#### 3.9.1 Design elements and corresponding statistical approaches

While researchers evaluating a PHI using NE methods may not have control over when, where and amongst whom an intervention has been implemented, they will likely have some control over choosing design elements of the evaluation that strengthen the credibility of the estimated effects. The control group within randomised studies provide the counterfactual outcome (i.e. what would have happened to programme participants, in the absence of the programme), whereas evaluations of NEs need to construct it, typically involving design elements with appropriate statistical approaches.

In order to create a credible design it is strongly recommended that researchers understand and justify the source of variation in the exposure to the intervention (item 9.1), which needs to have an exogenous element for credible causal inference (Meyer, 1995).

Different sources of variation can lead to three distinct designs and corresponding statistical and econometric approaches:

1. Designs using temporal and geographical variation: one-group before-after comparisons (Auer et al. (2016)); interrupted time series (Leyland et al., 2017); difference-in-difference (DiD) (Nandi et al. (2016)) and panel data methods (Pesko et al., 2016); synthetic control approach (Abadie et al., 2010). For before-after designs, identifying a potential control group and collecting frequent measurements of data on long pre-treatment time periods (Wagenaar et al., 2001) is a recommended best practice to offset the confounding role of potential anticipatory effects as well as other policies introduced at the same time (item 9.1.1).
2. Designs using individual level variation, where “nature” provides a variation in treatment assignment that resembles randomisation in the controlled situation of an actual experiment: IV eg. (Ichida et al., 2013; Yen et al., 2008); regression discontinuity design (RDD) (Andalón, 2011; Calonico et al., 2014; Imbens and Lemieux, 2008; Ludwig and Miller, 2007)
3. Designs aiming to construct a control group which best approximates an ideal randomised experiment: *matching* (e.g. propensity score matching and more recent covariate-balancing multivariate matching methods) (Caliendo and Kopeinig (2008); Stuart (2010); Zubizarreta, 2012) Melhuish et al. (2008)).

Often, more than one design is embedded in the evaluation (Craig et al., 2012), or different designs are combined. Exploring the sensitivity of cost-effectiveness results to approaches used to reduce the selection bias inherent to non-randomised studies allow examination of the sensitivity of economic evaluation to multiple sources of bias and strengthens the credibility of results (item 9.2). If all results are in the same direction with a similar magnitude, this gives the analyst increased confidence that the intervention had a true effect.

Most of the research designs previously described corresponds to well defined statistical and econometric methods (reviewed for example in (Athey and Imbens, 2017; Imbens and Rubin, 2015; Imbens and Wooldridge, 2009). For each design and statistical method, it is recommended to specify the list of potential confounders to control for, using substantive knowledge on the relationship between the intervention and the outcomes, and the mechanism of the assignment to the intervention (item 9.3).

In line with recommendations provided for item 2.3, if any concurrent intervention is identified, the causal effect should be interpreted with caution, in consideration of potential contaminating policies (item 9.4).

The design of a NE has implications for several aspects of the associated economic evaluation, especially the choice of an appropriate statistical method and the *interpretation* of the estimated effectiveness and cost-effectiveness parameters (e.g. IV and RDD only facilitate the estimation of local ATEs, which can be interpreted as incremental cost and incremental effectiveness parameters among a specific population).

Interpretation of the estimated effectiveness and cost-effectiveness results needs to be aligned to the estimated parameters (ATE, ATE on the Treated, Local ATE). When combining estimated parameters from different research design and data sources, or when reporting results using different statistical methods researchers need to transparently report which of these parameters are identified (item 9.5).

#### 3.9.2 Implementation of the statistical approaches in an economic evaluation setting

Existing literature regarding the implementation of some of the above listed statistical approaches (e.g. Stuart (2010) and Caliendo and Kopeinig (2008) Jacob et al. (2012)) rarely cover the specific challenges of economic evaluations, such as skewed and correlated cost and outcome data. Extensions of statistical approaches for the purposes of economic evaluation is a growing strand of methodological literature, for example, IV approaches have been extended to handle binary outcome data (Terza et al., 2008) as well as correlated cost and outcome data (DiazOrdaz et al., 2018).

Regression and matching methods can handle correlated data using a Bayesian framework (Nixon and Thompson (2005); Manca and Austin (2008)), as well as the non-parametric bootstrap (Sekhon and Grieve (2012) (Kreif et al. (2012); Kreif et al. (2013b)). Furthermore, flexible parametric and semiparametric approaches have been proposed to handle skewed cost distributions (Jones et al., 2015) and outcomes, e.g. quality of life data (Basu and Manca, 2012). For complex interventions, beyond the correlated costs and outcomes, a further challenge is handling potentially correlated multiple outcomes (Teixeira-Pinto and Normand, 2009).

If needed, clustering needs to be handled, e.g. using multilevel modelling or two-stage bootstrap, following recommendations that extend these methods for economic evaluations (Gomes et al., 2012). Given the importance of routinely collected data, as well as survey data in the evaluation of NEs, missing data is expected to be an important challenge for economic evaluation (See recommendations in Category 1)(Faria et al., 2014). The economic evaluation of PHIs alongside NEs should encompass methodologies to reduce selection bias into an econometric framework considering health economics-specific challenges (item 9.6).

### 3.10 Category 9: uncertainty & sensitivity analysis

The previous sections have outlined a range of sources of structural or methodological uncertainty (Briggs et al., 2006) when conducting economic evaluations alongside NEs. As for the RCT framework, all sources of uncertainty need to be identified, using recommended methods such as probabilistic sensitivity analysis, tornado diagrams (item 10.1), and reporting cost-effectiveness results according to different analytical choices (item 10.2). However, the researcher needs to address also additional sources of uncertainty specific to NEs that (item 10.3). Indeed, exploring sensitivity to several sources of unit costs (item 10.3.1) and assessing the sensitivity of potential spillovers (item 10.3.2) are common to RCT frameworks. However, exploring sensitivity to the different choice of comparators (item 10.3.3), designs (item 10.3.4), econometric approaches (item 10.3.5), unobserved confounding (item 10.3.6) and description of transfer payments (item 10.3.7) are additional sensitivity checks that needs to be performed in a NE framework.

## 4 Discussion

In the paper we have outlined the need for methodological guidance for conducting economic evaluations alongside NEs. Our guidance is based on the most recent methodological advances and has identified a set of best practices as a first step towards the development of a comprehensive framework. We have exemplified how the political and social aims inherent to PHIs and the selection bias inherent to the design of NEs pose unique challenges for health economic evaluations. For example, reliance on existing data sources does not allow the researcher to design data collection instruments to include ‘final’, utility-based outcomes, but offers advantages in terms of population representativeness. We have also highlighted that conducting economic evaluations of PHIs alongside NEs poses challenges which should be considered in the early design stage, in order to enhance the quality of the economic evaluation. For example, data linkage can overcome limitations of individual data sources by extending available data to also include a broad set of outcomes and costs. Similarly, long-term decision modelling can help linking short term, intermediate outcomes (e.g. children's birthweight) with longer term final outcomes (e.g. QALYs, life expectancy). Being able to address these challenges adequately and to robustly analyse NEs can be highly advantageous in settings where RCTs are unsuitable.

Our paper adds to available reporting guidelines (CHEERS, CONSORT, STROBE), recognizing the lack of a unique, comprehensive framework addressing the specific challenges of designing and conducting economic evaluations alongside NEs. We have focused on using NEs, rather than RCTs, to evaluate PHIs and highlighting the additional challenges arising from economic evaluation. Whilst recognizing that the available literature only provides a partial view of the challenges related to economic evaluation of PHIs alongside, the current work adapts the available literature, re-interpreting existing best practice in a systematic way.

This paper provides the first framework for conducting economic evaluations of PHIs alongside NEs, and offers a set of recommendations that can support researchers undertaking transparent and accurate evaluations, in line with existing NICE guidelines on economic evaluations of PHIs. Our proposed framework aims to improve and standardise the way economic evaluations of PHIs alongside NEs are conducted, providing a benchmark against which studies can be compared, thus has the potential to improve the overall quality and transparency of future evaluations. Furthermore, the set of guidelines we have developed is consistent with existing NICE guidance on conducting economic evaluations of PHIs, by recommending the use of a societal perspective, as well as alternative frameworks to CUA (e.g. CBA and CCA) to capture the battery of multi-sectoral outcomes of PHIs.

This paper also contributes to and expands existing studies (e.g. (Chalkidou et al., 2008; Lorgelly et al., 2010; Weatherly et al., 2009) focussing on and drawing out the benefits and challenges related to using NEs to evaluate PHIs, by highlighting methodological challenges such as matters of equity, the publicness (i.e. non excludability) of many PHIs, handling multiple outcomes and dealing with externalities. While this paper has focused on NE methods for evaluating PHIs, rather than clinical or healthcare interventions, further research should explore the challenges of conducting economic evaluations in those settings.

While the majority of methodological considerations specific to economic evaluation alongside NEs are not novel in themselves, our contribution lies in their collective use to deliver a framework for analysis to guide decision making. The paper has provided a starting point for a new and emerging research area, by identifying key areas for future research, including, but not limited to: developing well-established econometric methodologies to encompass approaches to reduce the selection bias inherent to NEs into an economic evaluation framework; developing a logic/conceptual modelling framework to simplify the intricacy inherent to economic evaluation of PHIs alongside NEs, guiding the researcher from early design to model development; explore suitability of alternative economic evaluation frameworks such as MCDA or CCA to evaluate PHIs in the presence of multiple, inter-sectoral outcomes; development of methodologies to incorporate long term inter-sectoral spillovers into economic evaluation.

## Appendix A Supplementary data

Supplementary Material_R2.docx Supplementary Material_R2.docx
