# Supplementary material for: A framework for conducting economic evaluations alongside natural experiments
Source: Soc Sci Med. 2019 Jan;220:353–61. doi: 10.1016/j.socscimed.2018.11.032 (PMC6323352; doi:10.1016/j.socscimed.2018.11.032)
Supplement: Supplementary Material_R2.docx [file mmc1.docx]

**Supplementary Material**

**Appendix A1 The Healthy Start Voucher case study**

1. **Background**

The Healthy Start Voucher (HSV) is a large-scale scheme that has been implemented nationally across the UK since November 2006. It replaced the Welfare Food Scheme (WFS), which was in place since 1940. It is a large scale nationally implemented program, involving the joint participation of health professionals (health visitors, nurses, midwifes) and private providers.

The Healthy Start scheme is designed to help low-income pregnant women and families with children under four to improve their nutrition by providing vouchers to buy liquid cow’s milk, plain fresh and frozen fruit and vegetables and infant formula milk. The general aim of HSV is to promote healthy lifestyle of pregnant women and children with a low socio-economic status- who are less likely to afford healthy food, and probably less aware of the benefits of a healthy diet- by changing their dietary habits and nutrition behaviour. The role of maternal nutrition in affecting child nutrition is crucial, and bad eating habits developed during childhood might perpetuate in the adulthood. Furthermore, a wide strand of literature has recognized the strong linkage existing between maternal nutrition during pregnancy and the onset of chronic diseases such as heart and cardiovascular diseases ([Barker, 1990](#_ENREF_2); [Mishra et al., 2004](#_ENREF_8); [Skinner et al., 2002](#_ENREF_10)).

The economic evaluation of the cost-effectiveness of HSV means tested voucher would contribute evidence regarding the incremental cost-effectiveness associated to an intervention directed towards improving nutrition of pregnant women and children. This may be useful to inform the policy maker aiming to change behaviour, improve health outcomes and reduce health inequalities. In particular, the benefits of the programme will be evaluated against potential costs and cost saving occurred, thus providing the policy maker with useful guidance on the value for money provided by HSV against alternative programmes. Table 1 below summarizes the key items related to the economic evaluation of HSV.

**Table 1 The Healthy Start Voucher (HSV) case study**

| Aims and objective | Evaluate effectiveness and cost-effectiveness of vouchers to buy liquid cow’s milk, plain fresh and frozen fruit and vegetables and infant formula milk. |
| --- | --- |
| Target population and subgroups | Women who are at least 10 weeks pregnant or has a child under 4, receive means-tested benefits, and have an income below 15,600.  Pregnant women under 18 are also eligible, even if they do not receive any benefit. |
| Data Sources | -Growing Up in Scotland (GUS), a representative sample of Scottish births, and it was carried out in 2011 (sweep 1) and 2013 (sweep 2), when the children were respectively 10 months and three years old.  GUS will be linked with six sources of administrative data: Hospital records (SMR01); Birth records (SMR02); A&E attendance; immunization records (SIR); Health visitor reports (CHSP); Dental inspection (MIDAS)  -Infant Feeding Survey (IFS), a representative sample of UK births in 2010 |
| Setting and location | Scotland-UK |
| Study perspective | NHS/Societal perspective |
| Comparators | 1. Exposed vs. eligible but not exposed 2. Exposed vs. nearly eligible 3. Eligible vs. nearly eligible |
| Time horizon | 10 months (GUS-Sweep 1); 3 years (GUS-Sweep 2) |
| Discount rate | 1.5%, in line with NICE public health economic evaluation methods guidelines([NICE, 2012](#_ENREF_9)). |
| Outcomes | Mothers outcome: Vitamin use in pregnancy  Child outcome: breastfeeding initiation; breastfeeding duration |
| Resource use and costs | Costs to the National Health system (hospitalization costs during pregnancy, related to the new born); health care services utilization related to the child; costs related to the usage of social services |
| Analytical methods | Propensity Score, Regression Discontinuity Design and intention to treat; Deterministic and probabilistic stochastic analysis; sensitivity analysis related to the main assumptions underlying propensity score and regression discontinuity |

The economic evaluation of HSV has been conducted alongside a natural experiment. The HSV has been used as a reference for piloting the checklist. This has led to critically assess its validity, which led to further improvements to the checklist, in an iterative process.

Table 2 below describes how each item of the checklist has been addressed by the economic evaluation of the checklist.

**Table 2 Implementation of the checklist in the HSV case study**

| **1. Data sources and measurement** |  |
| --- | --- |
| **1.1 The data used and the reason(s) why it has been chosen has been identified, stated and described in relation to:** |  |
| 1.1.1 All relevant intersectoral outcomes and costs being captured | *In the he HSV study the “Growing up in Scotland (GUS)” database, a representative sample of Scottish births, has been linked with six administrative databases, allowing the identification of a wide range of health and social care resource use, and improving the number of cost items collected as well as the quality of collected data. GUS contains information on HSV eligibility and uptake, and information about age and income, plus a wide range of socio-economic variables, and it covers the first 10 months of the children, plus the mother’s pregnancy. Linkage to CHSP, SMR01 and SMR02, MIDAS and SIRS has allowed to recover detailed information on a wide range of healthcare resources used by the mother and the child over a longer time horizon (6 years), thus providing information on whether the envisaged change in feeding and breastfeeding habits has led to better health outcomes in the long term .* |
| 1.1.2 implementation of the chosen statistical design | *GUS has allowed the identification of the two main control groups, associated with the chosen statistical approaches: PS control group: eligible, but not receiving HSV; RDD control group: nearly eligible (those who just miss the eligibility criteria for HSV, regarding income and age).* |
| **1.2 The application to routinely collected administrative data has been done on time to avoid delays in conducting economic evaluations (e.g. due to bureaucratic procedures, anonymization, privacy and confidentiality requirements).** | *Data linkage required an application to the Public Benefit and Privacy Panel to ensure the research is beneficial to the public and is carried out appropriately to avoid disclosure. Even if the application has been done on time, there have been delays in receiving the linked data.* |
| **1.3 The study recognize and address attrition and missing data and its consequences for the health economics analysis (bias)** | *A suitable method for missing data imputation will be chosen, according to the nature of missingness. No attrition bias is expected, given that only one wave of GUS will be used.* |
| **1.4 The study recognize and address measurement errors (e.g. due to discrepancies between the timing of the intervention and period of data availability) and its consequences for the health economics analysis (bias)** | *The linkage of GUS with administrative sources would give rise to additional reasons for missingness, given that routine data sources and clinical databases are often incomplete. A suitable method for missing data imputation will be chosen, according to the nature of missingness.* |
| **2. Setting and location** |  |
| **2.1 Setting and location are stated and explained in relation to social and political priorities** | *In the HSV example, the vouchers are directed towards deprived mothers and pregnant women, given the aim to improve the mothers’ and children’s health.* |
| **2.2 The source of secondary data that best meets the economic evaluation needs in terms of setting and location has been stated** | *The economic evaluation of the HSV was mainly conducted using Scottish data (GUS), even if it has been implemented throughout UK. The GUS survey was chosen as primary source of data since it allows for the identification of multiple intervention and comparison groups.* |
| **2.3 Concurrent interventions  have been:** |  |
| 2.3.1 Identified | *na* |
| 2.3.2 Tackled with appropriate statistical analysis (e.g. robustness checks; subsample analysis) | *na* |
| **2.4 Potential spillovers/externalities effects have been:** |  |
| 2.4.1 Identified through the usage of an economic evaluation logic model | *na* |
| 2.4.2 Addressed through appropriate sensitivity analysis | *na* |
| **3. Choice of comparators** |  |
| **3.1 The choice of comparators is justified in relation to reduction of selection bias due to non-randomisation, the unit of assignment (individual or aggregate) and data availability** | *In the HSV, two comparators were used: eligible, but not taking HSV (PS control group); nearly eligible, not taking HSV (RDD control group).* |
| **3.2 The existence of potential spillovers/crossovers has been considered in the choice of comparators** | *na* |
| **3.3 Multiple intervention/control groups have been used to examine sensitivity of the economic evaluation to multiple sources of bias** | *In the HSV study, three different methodologies to reduce the selection bias (Intention to treat (ITT), propensity score and Regression Discontinuity Design (RDD)) required the use of different intervention and control groups.* |
| **4. Subgroups** |  |
| **4.1 If equity concerns are included in the economic evaluation, subgroups are defined in relation to distributional concerns** | *The analysis of HSV will provide preliminary evidence of subgroups (defined by age or social deprivation) which are more likely to benefit from the intervention. However, given that the intervention is directed towards a relatively homogeneous population (low income women, in receipt of means tested benefit), it’s unlikely that it is going to generate unfair health inequalities.* |
| **4.2 Potential behavioral responses (e.g. ‘nudge effects’), have been identified and measured** | *In the HSV case study “nudge effects” has been not formally analysed, due to lack of data, but their existence has been accounted for. A similar case study (*[*Griffith et al., 2014*](#_ENREF_6)*) using longitudinal data on food purchase investigated whether consumption of fruit and vegetables changed after the introduction of the policy for “distorted” households (i.e. those who would spend less than the value of the vouchers on fruit and vegetables if given an equivalent cash benefit) an “inframarginal” households. They eventually found no difference between the two groups of households, thus not supporting the existence of a “nudge effect”.* |
| **5. Outcome** |  |
| **5.1 An economic evaluation model mapping routinely collected intermediate outcomes to QALYs has been developed, using additional evidence from systematic reviews to identify utility values.** | *In the HSV case study the primary outcomes in the evaluation are vitamin use in pregnancy and breastfeeding duration. These outcomes are intermediate, ‘proxy’ outcomes for final, longer term outcomes including quality of life, life expectancy and a range of non-health outcomes such as employment and income.  There is published evidence that breastfeeding duration is linked to longer term outcomes, including reduced risk of developing conditions including infectious diseases (*[*Duijts et al., 2009*](#_ENREF_4)*) and a lower relative risk of hospitalization for a range of individual illnesses reported in childhood(*[*Ajetunmobi et al., 2015*](#_ENREF_1)*). Hence, for the purposes of the HSV economic evaluation, if evidence of short term effectiveness of the intervention will be found, hazard ratios from such studies will be adapted to provide risk estimates for developing these conditions for the development of a long term economic evaluation model.* |
| **5.2 An economic evaluation framework such as CCA, CBA or MCDA has been chosen and justified** | *The cost per unit of increase in breastfeeding duration, initiation and vitamin intake will be analysed in a CBA framework. If the intervention will not show evidence of cost-effectiveness in the short term, a CCA framework will be used to provide descriptive evidence of any incremental effect or cost over the 6 years’ time horizon.* |
| **6. Costs** |  |
| **6.1 Costing has been done considering a societal perspective** | *Costing in the HSV has been done using a societal perspective, considering costs arising from the usage of social services, such as breastfeeding support groups, parentcraft, childcare. A list of resource use in the HSV case study is presented in Table 3.* |
| **6.2 When unit cost data associated to a specific resource use are not available, a decision rule (e.g. usage of the average unit cost of the most frequently used service) is explained and justified.** | *In the HSV case, the generic cost of ‘parentcraft’ has been attributed to a wide range of support-community programmes (PPP, mellow parenting), in the absence of a specific unit cost to be associated to each of these services.* |
| **6.3 When specific categories of resource use are not publicly available (e.g. HRG-specific length of stay), a decision rule (e.g. using the average cost/bed day) is explained and justified.** | *The administrative cost of running the program is not available in the HSV, and this is going to cause a downward bias in the estimated cost of the program.* |
| **6.4 The opportunity cost of transfer payments (i.e. transfer of resources from the government to beneficiaries, with a null net impact on society) has been identified and measured** | *The HSV, as the earlier HiP grant(*[*Dundas et al., 2014*](#_ENREF_5)*), was such a transfer payment with no net cost to society incurred through transferring the grant from government to individuals, apart from administrative costs(*[*Dundas et al., 2014*](#_ENREF_5)*). In the economic evaluation of HSV sensitivity analysis using the overall cost of 3.10/week HSV transfer payment has been done.* |
| **7. Time horizon** |  |
| **7.1 Linked data are adequate to capture the presence of long term effects** | *Linked data covering 6 years will allow considering potential beneficial effects (e.g. reduced hospitalizations) after the end of the intervention)* |
| **7.2 Appropriate discount rates, in line with the most up to date guidance have been applied** | *A discount rate of 3.5% will be applied. Sensitivity analysis using 1.5% will be done.* |
| **8. Inclusion of a logic model** |  |
| **8.1 A logic model has been developed, and it addresses:** |  |
| 8.1.1 Time horizon(e.g. long term effects) | yes |
| 8.1.2 possible subgroups effect | na |
| 8.1.3 externalities and spillovers | na |
| **9. Analytical methods** |  |
| **9.1 The researchers have justified the source of variation in the receipt of the intervention, choosing a design and a statistical approach which is appropriate in relation to that source of variation.** | *In the HSV case, a design aiming to construct a control group which best approximates an ideal randomised experiment (propensity score matching, IPTW) has been used, using eligible but not claiming HSV as control group. A design using individual level variation, where “nature” provides a variation in treatment assignment that resembles randomisation in the controlled situation of an actual experiment (‘fuzzy’ RDD ) has been also used, including ‘nearly eligible’ women (slightly below or above 18; income close to the 15,600 threshold) as control group.* |
| 9.1.1 If the study is a before after design frequent measurements of data on long pre-treatment time periods have been collected | na |
| **9.2 Multiple statistical designs have been employed to examine the sensitivity of economic evaluation to multiple sources of bias** | *The HSV evaluation protocol proposes both a RDD and propensity score matching approach to create multiple comparison groups for robustness in evaluation.* |
| **9.3 The list of potential confounders has been presented** | *The HSV protocol states a list of potential confounders, including demographic (age, gender, civil status) and socioeconomic characteristics (Deprivation index..)* |
| **9.4 Causal effects have been interpreted considering potential contaminating policies** | **na** |
| **9.5 The interpretation of the estimated effectiveness and cost-effectiveness is in line with the estimated parameter** | *ATE estimated using the propensity score (intervention group: eligible & in receipt of HSV; control group: eligible, but not receiving HSV) measures the incremental effect (cost) of HSV if all women would take HSV (vs. not taking HSV); ITT estimated using the propensity score (intervention group: eligible; control group: not eligible) measures the incremental effect (cost) of being made eligible for HSV (regardless of uptake) LATE estimated using fuzzy RDD measures the incremental effect (cost) of HSV for women who actually took HSV ('compliers') (this is the effect of HSV for the subsample of women near the eligibility threshold)* |
| **9.6 The methodologies to reduce selection bias have been incorporated into an econometric framework, considering health economics-specific challenges (i.e. skewed outcome and cost data, correlated outcome and cost data).** | *Cost-effectiveness analysis on a sample matched with propensity score (*[*Manca & Austin, 2008*](#_ENREF_7)*); usage of the framework developed by (*[*DiazOrdaz et al., 2017*](#_ENREF_3)*) to handle correlated cost and outcome data within a fuzzy RDD framework.* |
| **10. Uncertainty and sensitivity analysis** |  |
| **10.1 All sources of uncertainty have been identified using appropriate methods (e.g. probabilistic sensitivity analysis; tornado diagrams)** | yes |
| **10.2 Cost-effectiveness results according to the different analytical choices have been reported** | **yes** |
| **10.3 Sensitivity analysis has been done in relation to:** |  |
| 10.3.1 Assumptions made in relation to unit cost | yes |
| 10.3.2 Potential spillovers | na |
| 10.3.3 Comparators | yes |
| 10.3.4 Different designs | yes |
| 10.3.5 econometric methodology chosen | yes |
| 10.3.6 Unobserved confounding | yes |
| 10.3.7 Transfer payments and administrative costs | yes |

**Table 3: Resource use in the HSV case study**

| **Cost Item** | **Type of variable** | **Unit of cost measure** |
| --- | --- | --- |
| **Variables only available in GUS** | | |
| First ante-natal appointment with: family doctor, nurse, consultant etc. | dummy yes/no | Visits |
| Attendance of antenatal class | dummy yes/no | Visits |
| Has received breastfeeding advice from health visitor/midwife/National Childbirth Trust etc | dummy yes/no | Visits |
| Took part in support-community programmes (PPP, mellow parenting) in the past 12 months | dummy yes/no | Visits |
| Use of programmes to help parents and their families (ccare, link website/phoneline, parentline scotland, childsmile...) | dummy yes/no | Visits |
| Use of web-based helping services (mumsnet, facebook, bebo, ivillage) | dummy yes/no | Visits |
| **Variables available in GUS and in linked datasets** | | |
| Type of delivery | categorical var | Cost/intervention |
| **The Scottish Morbidity records database-Birth records (SMR02)** contains more detailed information on type of delivery | | |
| Frequency of health visitor in the first 3 months since baby was born | categorical var | Visits |
| **The Child health system programme pre-school (CHSP)** contains more detailed information on health visitor records: hearing screening, health visitor first visit report, 6-8 week review, orthoptist vision screening, recall review | | |
| no. of times the child went to hospital for health problems and illness (excluding accidents and injuries) | count | Cost/day |
| **The Scottish Morbidity records database (SMR01)** contains more detailed information on detailed information on : specialty/discipline, management of patient (inpatient, outpatient, day case) | | |
| **Variables only available in linked datasets** | |  |
| ***SMR02 (Hospital inpatient/day case admission maternity obstetric records)*** | | |
| length of stay | continuous | Cost/day |
| labour record |  | Cost/specific procedures |
| ***A&E ATTENDANCE*** |  |  |
| Length of stay | continuous | Cost/day |
| ***SIR (IMMUNIZATION RECORDS)*** |  |  |
| whether had a vaccine and type of vaccine | dummy/categorical | Dose |
| ***MIDAS (DENTAL INSPECTION)*** |  |  |
| type and no. of treatment | count | Visit |

**Appendix A2 Targeted review**

The search was performed over a broad set of electronic resources (e.g. Web of Science; Embase). Furthermore, we used hand searching from Google Scholar, reviewed key journals and critically reviewed reference lists of key papers to extract relevant articles. The search was limited to articles written in English, without any time limit. The following keywords were used for the search: ("cost-effectiveness" OR "cost effectiveness "OR "economic evaluation" OR "cost-benefit" OR "cost benefit") and (“natural experiment" OR "quasi experiment" OR "quasi-experiment" OR "observational design" OR "non randomized" OR "non-randomized" OR "regression discontinuity" OR "Difference in difference" OR "propensity score”)

In order to restrict the scope of the analysis to a well-defined field of applicability we deliberately exclude economic evaluation of PHIs using frameworks different from NEs (e.g. RCTs), or NEs not regarding PHIs. We restrict the analysis to natural and quasi-experiments since we consider a non- randomised intervention, excluding the observational studies where no intervention takes place.

The final outcome of the search has been decided after a common assessment made by all the authors during all the steps of the review. This discussion has informed sampling and selection of material.

The iterative process has followed the following steps:

1. Initial research question scopes initial literature search:
   1. Natural experiments
   2. Economic evaluations
2. Refine the research question
   1. Natural experiments, excluding observational studies
3. New search with refined research question
4. Refine the research question
   1. Exclude studies not regarding PHIs
5. Final scope of the review

Reference List

Ajetunmobi, O.M., Whyte, B., Chalmers, J., Tappin, D.M., Wolfson, L., Fleming, M., et al. (2015). Breastfeeding is associated with reduced childhood hospitalization: evidence from a Scottish Birth Cohort (1997-2009). *The Journal of pediatrics,* 166, 620-625. e624.

Barker, D.J. (1990). The fetal and infant origins of adult disease. *BMJ: British Medical Journal,* 301, 1111.

DiazOrdaz, K., Franchini, A., & Grieve, R. (2017). Methods for estimating complier average causal effects for cost‐effectiveness analysis. *Journal of the Royal Statistical Society: Series A (Statistics in Society)*.

Duijts, L., Ramadhani, M.K., & Moll, H.A. (2009). Breastfeeding protects against infectious diseases during infancy in industrialized countries. A systematic review. *Maternal & child nutrition,* 5, 199-210.

Dundas, R., Ouedraogo, S., Bond, L., Briggs, A.H., Chalmers, J., Gray, R., et al. (2014). Evaluation of health in pregnancy grants in Scotland: a protocol for a natural experiment. *BMJ Open,* 4, e006547.

Griffith, R., Scholder, S.v.H.K., & Smith, S. (2014). *Getting a Healthy Start?: Nudge Versus Economic Incentives*: Centre for Market and Public Organisation.

Manca, A., & Austin, P.C. (2008). Using propensity score methods to analyse individual patient level cost effectiveness data from observational studies. *The University of York: Health Economics and Data Group Working Paper,* 8, 20.

Mishra, G., Prynne, C., Paul, A., Greenberg, D., & Bolton-Smith, C. (2004). The impact of inter-generational social and regional circumstances on dietary intake patterns of British adults: results from the 1946 British Birth Cohort. *Public health nutrition,* 7, 737-744.

NICE. (2012). Methods for the development of NICE public health guidance.

Skinner, J.D., Carruth, B.R., Bounds, W., & Ziegler, P.J. (2002). Children's food preferences: a longitudinal analysis. *Journal of the American Dietetic Association,* 102, 1638-1647.
